# Understanding bacterial pathogenicity: a closer look at the journey of harmful microbes

**DOI:** 10.3389/fmicb.2024.1370818

**Published:** 2024-02-20

**Authors:** Jyoti Soni, Sristi Sinha, Rajesh Pandey

**Affiliations:** ^1^Division of Immunology and Infectious Disease Biology, Integrative Genomics of Host Pathogen Laboratory, Council of Scientific & Industrial Research-Institute of Genomics and Integrative Biology, New Delhi, India; ^2^Academy of Scientific and Innovative Research, Ghaziabad, India; ^3^School of Biosciences and Technology, Vellore Institute of Technology University, Vellore, India

**Keywords:** bacteria, host susceptibility, immune response, AMR, disease severity

## Abstract

Bacteria are the most prevalent form of microorganisms and are classified into two categories based on their mode of existence: intracellular and extracellular. While most bacteria are beneficial to human health, others are pathogenic and can cause mild to severe infections. These bacteria use various mechanisms to evade host immunity and cause diseases in humans. The susceptibility of a host to bacterial infection depends on the effectiveness of the immune system, overall health, and genetic factors. Malnutrition, chronic illnesses, and age-related vulnerabilities are the additional confounders to disease severity phenotypes. The impact of bacterial pathogens on public health includes the transmission of these pathogens from healthcare facilities, which contributes to increased morbidity and mortality. To identify the most significant threats to public health, it is crucial to understand the global burden of common bacterial pathogens and their pathogenicity. This knowledge is required to improve immunization rates, improve the effectiveness of vaccines, and consider the impact of antimicrobial resistance when assessing the situation. Many bacteria have developed antimicrobial resistance, which has significant implications for infectious diseases and favors the survival of resilient microorganisms. This review emphasizes the significance of understanding the bacterial pathogens that cause this health threat on a global scale.

## Introduction

“The enemy was the microbial world, and over the centuries, it has killed more people than all of man's wars combined.”

—Tess Gerritsen, Gravity

**Figure 1:**
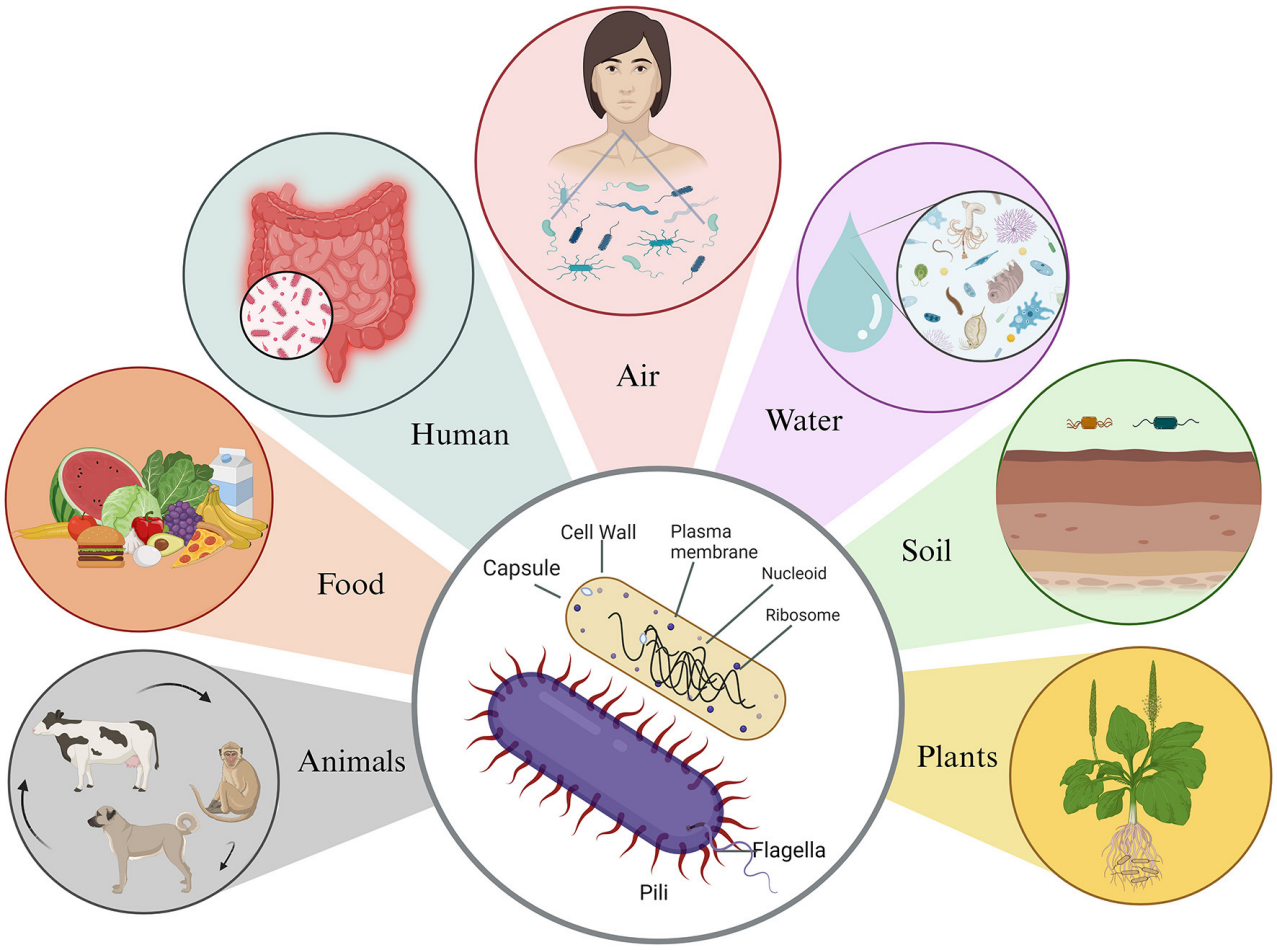
Basic structure of bacteria and their associated environmental reservoirs. Bacteria possess a simple body structure without a true nucleus or membrane-bound organelles. Their basic structure comprises the cell wall, which offers structural support and protection, and the plasma membrane, which surrounds the cytoplasm and distinguishes it from the external environment. In addition, some bacteria possess a protective outer layer known as the capsule, which aids them in evading the host's immune system. Bacteria are commonly found in various environments, such as animals, food, humans, air, water, soil, and plants.

Microbes are tiny and trillion can make us sick or can help us stay healthy (Malla et al., 2018). Of all microbes, the most abundant are bacteria, which are ubiquitous in nature and found in every conceivable habitat, from the soil beneath our feet to the depths of the Earth's crust and even in extreme environments like acidic hot springs and areas contaminated with radioactive waste (Bardgett and van der Putten, 2014; Thakur et al., 2022; Rappaport and Oliverio, 2023). Remarkably, bacteria not only coexist with humans and animals but also live within them (Figure 1) (Ursell et al., 2012). These microorganisms showcase incredible diversity in terms of size and shape. Bacterial cells typically range from 0.5 to 5.0 μm in length, with some exceptions such as the giant *Thiomargarita namibiensis*, which can be 50 times larger than most known bacteria (Levin and Angert, 2015; Miller and Murray, 2023). They come in various shapes, including spherical (cocci), rod-shaped (bacilli), slightly curved rods or comma-shaped (vibrio), spiral-shaped (spirilla), and tightly coiled (spirochaetes). In addition to their physical diversity, bacteria exhibit metabolic versatility (Young, 2006). Their metabolism can be categorized based on their energy source, electron donors, and carbon source. Some, known as phototrophic bacteria, harness energy from light through photosynthesis. Other, chemotrophic bacteria, break down chemical compounds through oxidation, using different electron donors and acceptors in redox reactions (Deusenbery et al., 2021).

These can be further classified as lithotrophs, which utilize inorganic compounds like hydrogen, or organotrophs, which rely on organic compounds. Some bacteria are heterotrophs, obtaining carbon from other organic sources, while others, like blue-green algae, are autotrophs, fixing carbon dioxide for growth (Legendre and Troussellier, 1988; Eiler, 2006). In unique situations, methanotrophic bacteria can utilize methane as both an energy source and a substrate for carbon anabolism. This interconnected and diverse world of bacteria underscores their significance in Earth's ecosystems and their intriguing role in the circle of life (Ahmadi and Lackner, 2024). These bacteria have proven good and bad impacts on human health. While the majority of these are quite helpful a few of these causes' death alarming diseases (Thakur et al., 2019). A fine example of bacterial pathogenesis is Mycobacterium tuberculosis infection (Schmidt and Hensel, 2004). When a person gets infected with *M. tuberculosis* for the first time, the bacteria multiply in the lungs and can spread to nearby lymph nodes and even other parts of the body (Bussi and Gutierrez, 2019). Surprisingly, this initial infection usually doesn't cause any symptoms in adults. However, the person's immune system steps in to control the bacteria's growth and spread (Glickman and Jacobs, 2001). Some bacteria specifically show specificity to the organs and various tissues as in the case of *Neisseria meningitidis* (Rouphael and Stephens, 2012). Other bacteria can be found everywhere in the host body, such as *Staphylococcus aureus* found in most of the tissues from skin to the bloodstream and in various organs (Sender et al., 2016). Despite the strong immune response, the bacteria are rarely eliminated. Instead, *M. tuberculosis* has a remarkable ability to enter a dormant phase, during which the person doesn't show any symptoms of tuberculosis but still carries the bacteria (Kiazyk and Ball, 2017). Also, the degree of infection caused by bacteria can vary from person to person, as it is influenced by the host factors such as host genetics, lifestyle, age, previous infections, nutrition, and environment (Ogunrinola et al., 2020; Hou et al., 2022). The host's susceptibility is also influenced by the surroundings. Environmental pollutants, chemicals, and air pollution all impair the body's ability to fight off bacterial infections (Kraemer et al., 2019). Unlike infectious diseases caused by viruses and parasites, antimicrobial resistance in bacteria is an emerging issue with severe repercussions (Muzyka, 1996).

## Overview of bacterial classification

### Based on staining

Bacteria are a diverse group of microorganisms and are classified based on various factors, including their structure and function. Staining is a useful method for categorizing bacteria, and it results in two main groups: Gram-positive and Gram-negative bacteria (Liu et al., 2021).

Gram-positive bacteria, like *Staphylococcus aureus, Staphylococcus epidermis, Streptococcus pneumoniae, Clostridium*, and *Bacillus anthracis*, have a single, thick peptidoglycan layer in their cell walls, which makes them “monoderm” (Desvaux et al., 2018; Chateau et al., 2020; Nikolic and Mudgil, 2023). On the other hand, Gram-negative bacteria, such as *E. coli, Klebsiella, Pseudomonas aeruginosa, H. pylori*, and *P. mirabilis*, have a thinner layer of peptidoglycan in their cell walls, which is surrounded by an outer membrane of lipopolysaccharides, making them “diderms” (Moyes et al., 2009).

**Figure 2:**
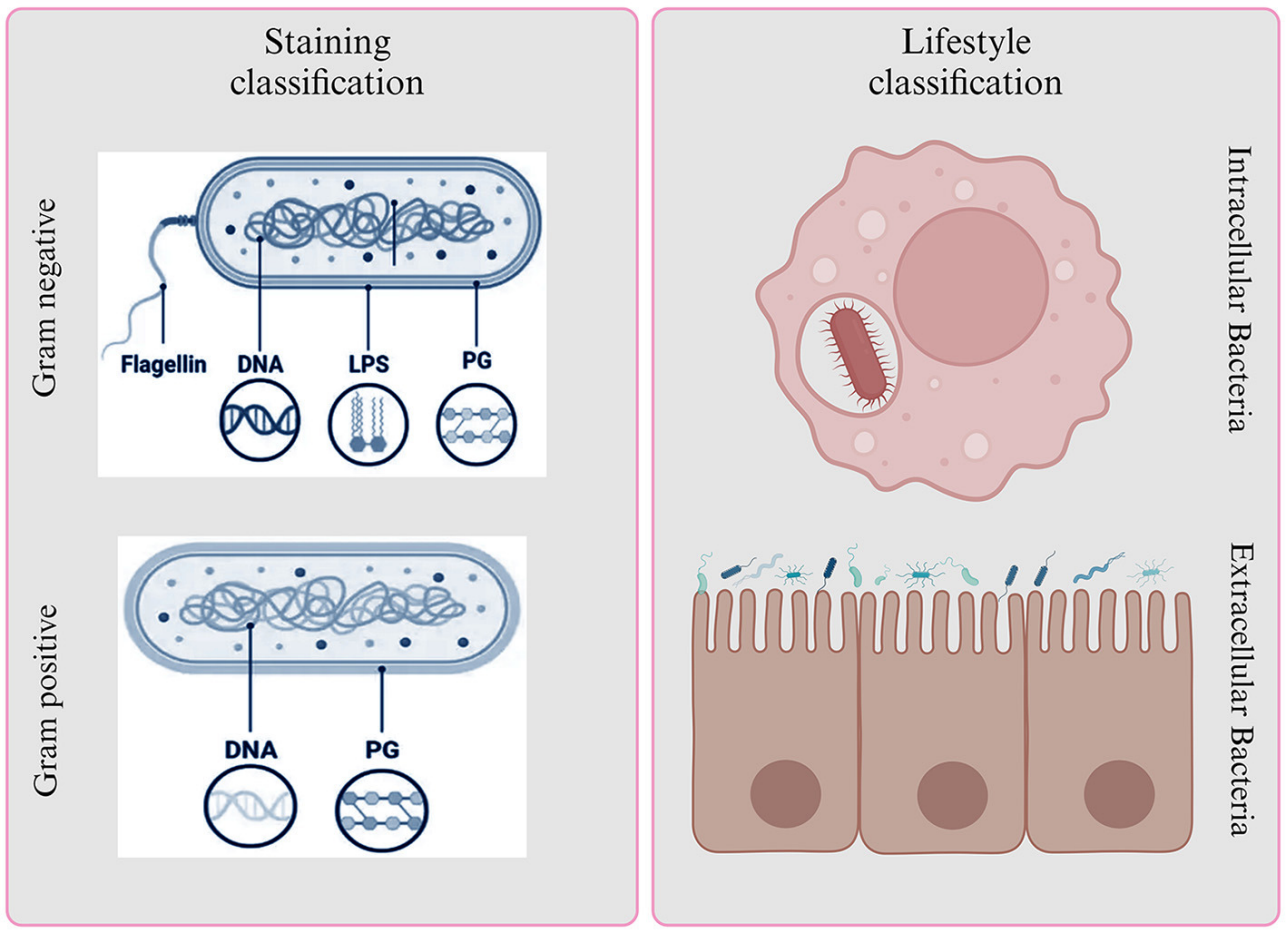
Bacterial classification based on Gram staining and lifestyle. It provides insights into their structural characteristics and interactions with host environments.

Gram-positive bacteria appear bluish-purple because their thick peptidoglycan layer retains the crystal violet and iodine, preventing it from washing off. Gram-negative bacteria, however, stain red because their thin peptidoglycan layer cannot hold onto the crystal violet and iodine, and the safranine counterstain takes over (Tripathi and Sapra, 2023). In certain conditions, Gram-positive bacteria can form spores as a survival mechanism when exposed to environmental stress, such as a lack of carbon and nitrogen. These spores help the bacteria endure and potentially cause infections (Becerra et al., 2016). In contrast, Gram-negative bacteria have an additional, permeable outer membrane that requires transport mechanisms across it. This outer membrane contains endotoxins, which contribute to the survival of Gram-negative bacteria. However, Gram-negative bacteria are more dangerous than their Gram-positive counterparts due to their formidable defenses (Hoerr et al., 2012; Ramachandran, 2014). They possess an outer membrane that acts as a barrier, efflux pumps that actively remove antibiotics, and produce enzymes like beta-lactamases that can disarm common drugs (Munita and Arias, 2016). These bacteria can form biofilms, making them resistant to treatment, and can change surface structures to evade the immune system. What's particularly concerning is their propensity for multi-drug resistance, rendering many antibiotics ineffective (Figure 2) (Becerra et al., 2016).

### Based on lifestyle

Based on bacterial lifestyle and environmental conditions they can be either intracellular or extracellular. Intracellular bacteria are pathogenic microorganisms capable of establishing a relationship with a susceptible host by multiplying within host cells (Casadevall, 2008). Examples include *Brucella abortus, Listeria monocytogenes, Chlamydia trachomatis, Coxiella burnetiid, Mycobacterium tuberculosis*, and *Salmonella enterica* (Drevets et al., 2004; Guo et al., 2023). These pathogens require specific host cells that support their intracellular growth conditions (Ray et al., 2009). They can be categorized as obligate, unable to multiply outside host cells, or facultative, with the ability to multiply both inside and outside cells. Infections caused by intracellular bacteria include brucellosis, listeriosis, tuberculosis, and salmonellosis (Silva, 2012; Jiao et al., 2021). These pathogens use various pathways to enter host cells, such as macrophages, phagocytes, epithelial and endothelial cells, and hepatocyte (Kaufmann, 1993; Thakur et al., 2019). Some may also transmit intercellularly without an extracellular phase. Intracellular bacteria must evade the host's immune response to survive and replicate within mononuclear phagocytes (Kaufmann, 1993; Silva, 2012).

On the other hand, extracellular bacteria, including *Staphylococcus aureus, Streptococcus pyogenes, Pseudomonas aeruginosa*, and *Escherichia coli*, exist outside host cells (Belon and Blanc-Potard, 2016). They cause conditions like wound infections, osteomyelitis, scarlet fever, certain types of pneumonia, and urinary tract infections. These pathogens typically multiply in extracellular spaces, such as mucosal surfaces, vascular and lymphatic fluids, and body cavities (Britton and Saunders, 2010; Sansonetti and Puhar, 2010; Mir et al., 2022). Occasionally, they may be found within phagocytes, which are part of the host's defense system. Immune evasion by extracellular bacteria involves mechanisms like humoral immunity and phagocytes, promoting their multiplication outside cells (Pieters, 2001; Schechter et al., 2019). Some extracellular bacteria can behave like intracellular ones in the early stages of infection, including *Staphylococcus aureus, Streptococcus pyogenes, Streptococcus pneumonia, Bacillus anthracis, Escherichia coli, Bordetella pertussis*, and *Helicobacter pylori*, surviving or replicating inside cells *in vitro* and within amoebas (Riffaud et al., 2023). Some extracellular bacteria may not penetrate body tissues but attach to epithelial surfaces and release toxins to cause disease (Schechter et al., 2019).

### Bacterial infection and pathogenesis

**Figure 3:**
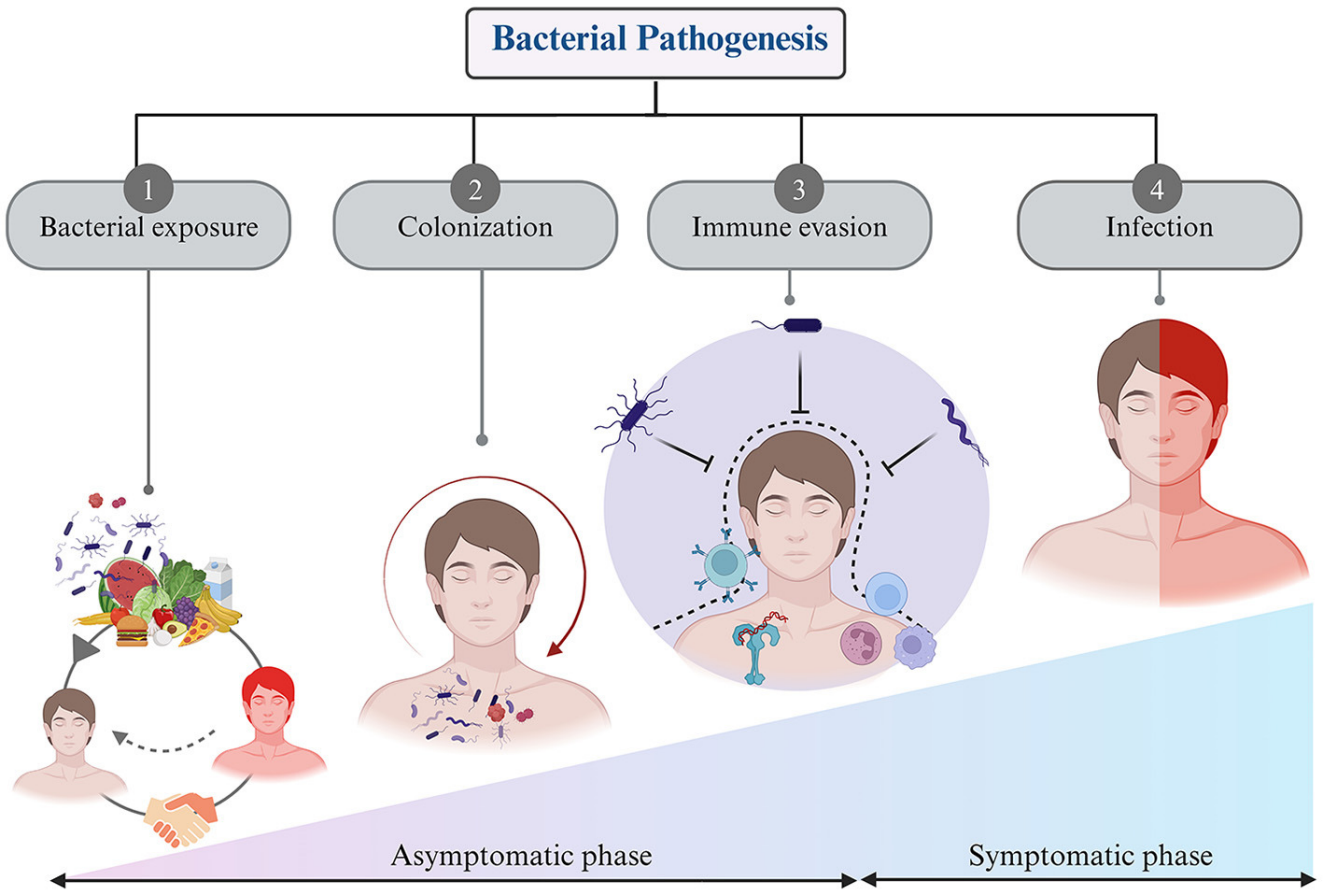
Steps of bacterial pathogenesis: (1) Bacterial exposure to host through air, food, infected person, and environment, (2) Colonization of the bacteria inside host within specific organs, tissues, and cells, (3) Evasion of immune response through diverse strategies, and (4) generating negative impact on host by causing infection.

Pathogenic bacteria are a subset of bacteria which can cause diseases in humans, while most bacteria are harmless or beneficial. The human body hosts thousands of gut flora bacteria (Rolhion and Chassaing, 2016). It encounters various bacteria, including commensals and saprophytes. Defense mechanisms provide innate resistance to microbial invasion. Pathogenic bacteria have evolved mechanisms to overcome this defense and invade the body. Infections usually occur when the body's defense are compromised, due to factors like trauma or underlying diseases. The pathogenicity of bacterial species is determined by their ability to cause disease and symptoms, varying in degree based on their virulence (Shapiro-Ilan et al., 2005). Some bacteria exist in avirulent forms and have little impact on health, but those actively transcribing virulence genes within a host cell can pose significant problems. Virulence factors encompass toxins (such as enterotoxins), surface coats, and surface receptors that bind to host cells. As previously mentioned, bacteria play a crucial role in the healthy human body, and any disruption in their balance can lead to disease (Farrell et al., 2021). Bacteria and their host mutually influence each other's activities and functions. Pathogenicity depends on the pathogen's resistance to host defense mechanisms and the host's susceptibility to bacterial virulence factors (Casadevall and Pirofski, 2001). The process of bacterial pathogenesis involves several key steps: contact, colonization, invasion, evasion of host defense, and ultimately infection (Chaffey et al., 2003) (Figure 3).

- *Bacterial exposure* encloses encounters with bacteria via diverse techniques, including contact with surfaces or objects bearing bacterial contamination, the ingestion of food or water harboring bacterial agents, inhalation of airborne bacterial particulates, or direct physical contact with individuals manifesting bacterial infections. Bacterial exposure is a ubiquitous phenomenon in daily existence and manifests the potential for both advantageous and detrimental consequences (Sharma and Gilbert, 2018). On occasions, exposure to commensal or probiotic bacteria can confer immunomodulatory benefits, thereby fortifying the host's immune system or yielding other advantageous effects. Conversely, exposure to pathogenic bacterial strains with virulent attributes can precipitate infectious ailments and maladies (Mazziotta et al., 2023).
- *Bacterial colonization*—Upon encountering a host, certain bacteria exhibit the capability to establish an enduring presence within the host's organism. Bacterial colonization is the process of bacteria initially attaching to a surface or host, then multiplying and forming a stable community (Barron and Young, 2022). This progression is influenced by factors such as the unique traits of the bacteria, the properties of the surface, and environmental conditions (Gollan et al., 2019).
- *Immune system escape*—Bacteria exhibit a remarkable ability to evade immune surveillance a pivotal strategy for their survival within the host environment. This adaptive skillset plays a crucial role following their colonization of the host (Kahn et al., 2002; Finlay and McFadden, 2006). These immune evasion strategies include several sophisticated steps, including inhibiting immune-related signaling pathways, concealing within various host cells, disrupting phagosomes, deactivating reactive oxygen species, and modulating the host's immune response by altering the molecular patterns on their outer surfaces (Van Avondt et al., 2015; Rana et al., 2023). These molecular modifications serve as a camouflage, rendering these bacteria less recognizable to the host's immune receptors. By doing so, bacterial pathogens avoid detection, enabling them to persist and thrive within the host's body (Finlay and McFadden, 2006). The mastery of these intricate camouflage and precision weaponry techniques by bacterial pathogens significantly complicates the development of novel vaccines and innovative treatments. In essence, the bacterial world's ability to navigate and manipulate the host's immune defenses represents a formidable challenge, requiring ingenious approaches to counter their crafty strategies and advance medical interventions (Table 1).
- *Bacterial infection*—Infectious diseases impose an enormous global burden, affecting public health systems and economies worldwide. Although whether the bacteria can cause symptomatic infection or not depends on its virulence ability as stated above. Majority of the bacteria uses the host for their replication and nutrition purpose without causing much harm (Grant and Hung, 2013; Kiazyk and Ball, 2017). Bacterial diseases can vary depending on the type of organism and the immune system of the host. Numerous pathogenic bacteria display different virulence factors, which can result in a variety of infection signs and symptoms (Fierer et al., 2017). Certain bacteria develop a mutually beneficial relationship with their host and are therefore harmless and possibly even beneficial, but virulent species, such as *Vibrio cholera*, can infect humans and cause serious health problems or even death (Høiby et al., 1986).

**Table 1:** Some bacteria-induced tissue-specific diseases and evasion strategies.

**S. No**	**Bacterial species**	**Bacteria lifestyle**	**Infection site**	**Disease**	**Evasion strategy**	**References**
1.	*Streptococcus pneumoniae*	Intracellular	Upper respiratory tract	Sinusitis, pneumonia, osteomyelitis, septic arthritis	Inhibits neutrophil phagocytosis	Hyams et al., 2010; Subramanian et al., 2019
2.	*Neisseria meningitidis*	Intracellular (facultative)	Nasopharynx	*Neisseria meningitidis*	Dysregulation of nutritional immunity	Coureuil et al., 2019; Mikucki et al., 2022
3.	*Pseudomonas aeruginosa*	Extracellular/Intracellular	Skin and soft tissue	Endophthalmitis, endocarditis, meningitis, pneumonia	Biofilm generation	Kumar et al., 2022; Kroken et al., 2023
4.	*Mycobacterium tuberculosis*	Intracellular	Lungs	Tuberculosis	Ability to persist in macrophages	Parbhoo et al., 2022; Toniolo et al., 2023
5.	*Listeria monocytogenes*	Intracellular (facultative)	Brain, blood stream	Listeriosis	Escape in cytosol	Osek and Wieczorek, 2022
6.	*Salmonella enterica* Typhi	Intracellular	Gastrointestinal tract	Typhoid fever	Stops fusion lysosome with autophagosome	Wang et al., 2021; Carey et al., 2023
7.	*Rickettsia* spp.	Intracellular	Bones	Rocky Mountain spotted fever, rickettsia pox	Escape into cytosol	Voss et al., 2021
8.	*Chlamydia* spp.	Intracellular	Cervix, urethra, throat and eyes.	Pelvic inflammatory disease (PID)	Degradation of host proteins and deactivation of neutrophils.	Sarkar et al., 2015; Rajeeve et al., 2018
9.	*Anaplasma phagocytophilum*	Intracellular	Neutrophils, granulocytes	Anaplasmosis	Inhibits autophagosome-lysosomal fusion.	Dumler et al., 2005
10.	*Brucella* spp.	Intracellular (facultative)	Liver, heart and central nervous system	Brucellosis	Inhibit fusion with host lysosomal compartment	Jiao et al., 2021

### Host susceptibility

**Figure 4:**
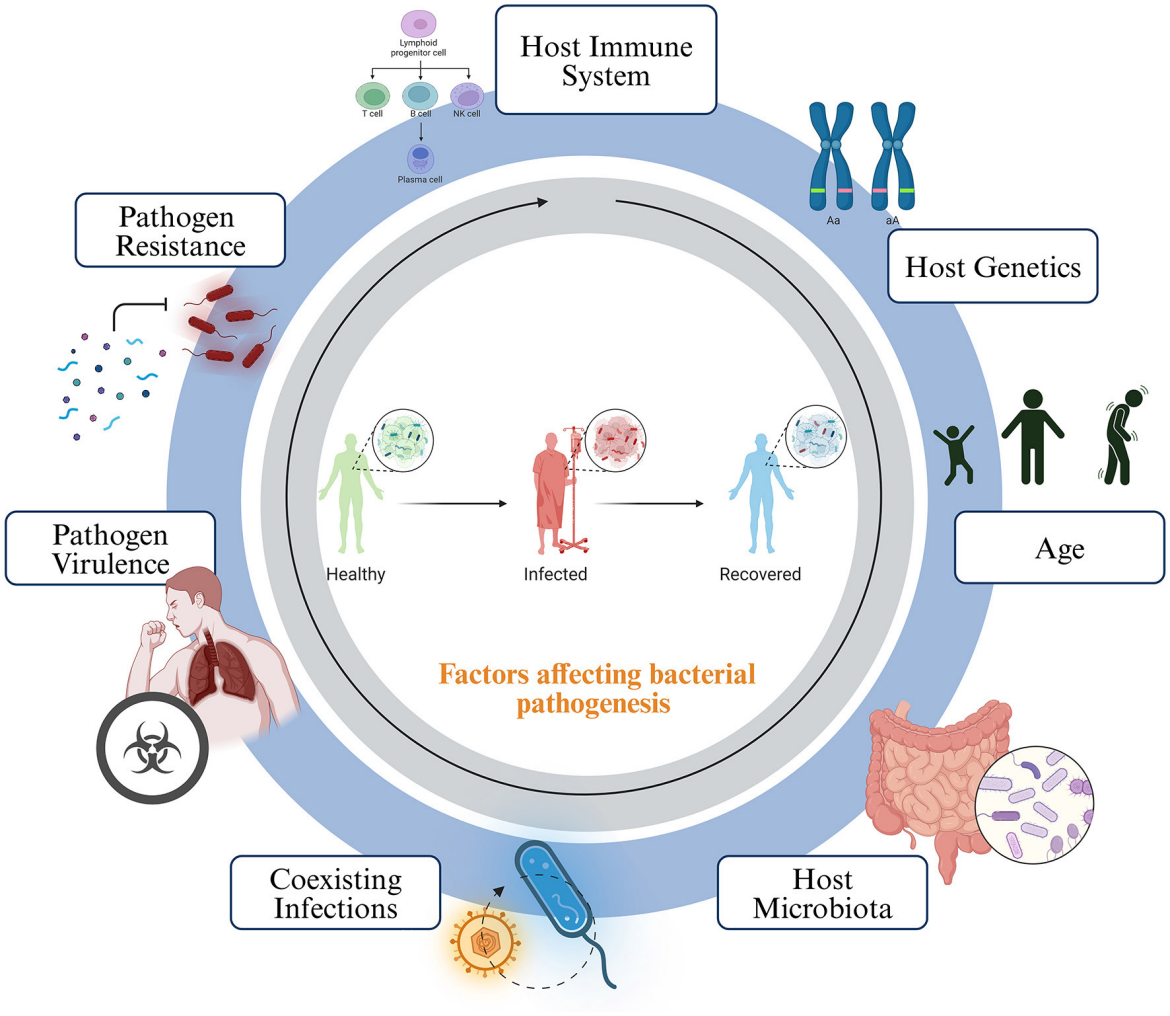
Host factors affecting the growth and pathogenesis of bacteria. The disease outcome is heavily influenced by the interactions between the host and the microbes. This interaction can be determined by several factors, such as the host's immune system, genetic makeup, age, natural microbiota, co-existing asymptomatic infections, pathogen virulence, and antimicrobial resistance.

Host susceptibility to bacterial infections is a complex interplay of various factors. The host's immune system stands at the forefront, with a strong and effective defense crucial in preventing or controlling bacterial invaders (Dropulic and Lederman, 2016). The overall health and immune status of the host are critical in comprehending the host susceptibility. Conditions like malnutrition, chronic illnesses, or immunosuppressive medications can increase susceptibility. Genetic factors also play a role, as some individuals may have genetic variations that influence their vulnerability to specific infections. Age is a significant determinant, with children and the elderly often more susceptible (Burgner et al., 2006). The microbiota inhabiting the host can act as a protective barrier, competing for resources and producing antimicrobial substances. Anatomical and physiological factors are also influential; structures like cilia and mucus in the respiratory tract and the stomach's acidity can deter bacterial growth (Figure 4).

Prior exposures and immunity, acquired through previous infections or vaccinations, can reduce susceptibility to subsequent infections. The environment in which the host lives and works matters, with sanitation, access to clean water, and exposure to contaminated surfaces or vectors affecting the likelihood of infection (Tomalka et al., 2022). Coexisting infections can weaken the host's immune system or create favorable conditions for other bacterial pathogens. The attributes of the bacterial pathogen itself are pivotal. Virulence factors and antibiotic resistance can enhance a pathogen's ability to cause infection (Pan et al., 2020). Understanding these determinants of susceptibility is essential for developing effective strategies for the prevention and treatment of bacterial infections (Fasciana et al., 2019).

### Immune evasion

**Figure 5:**
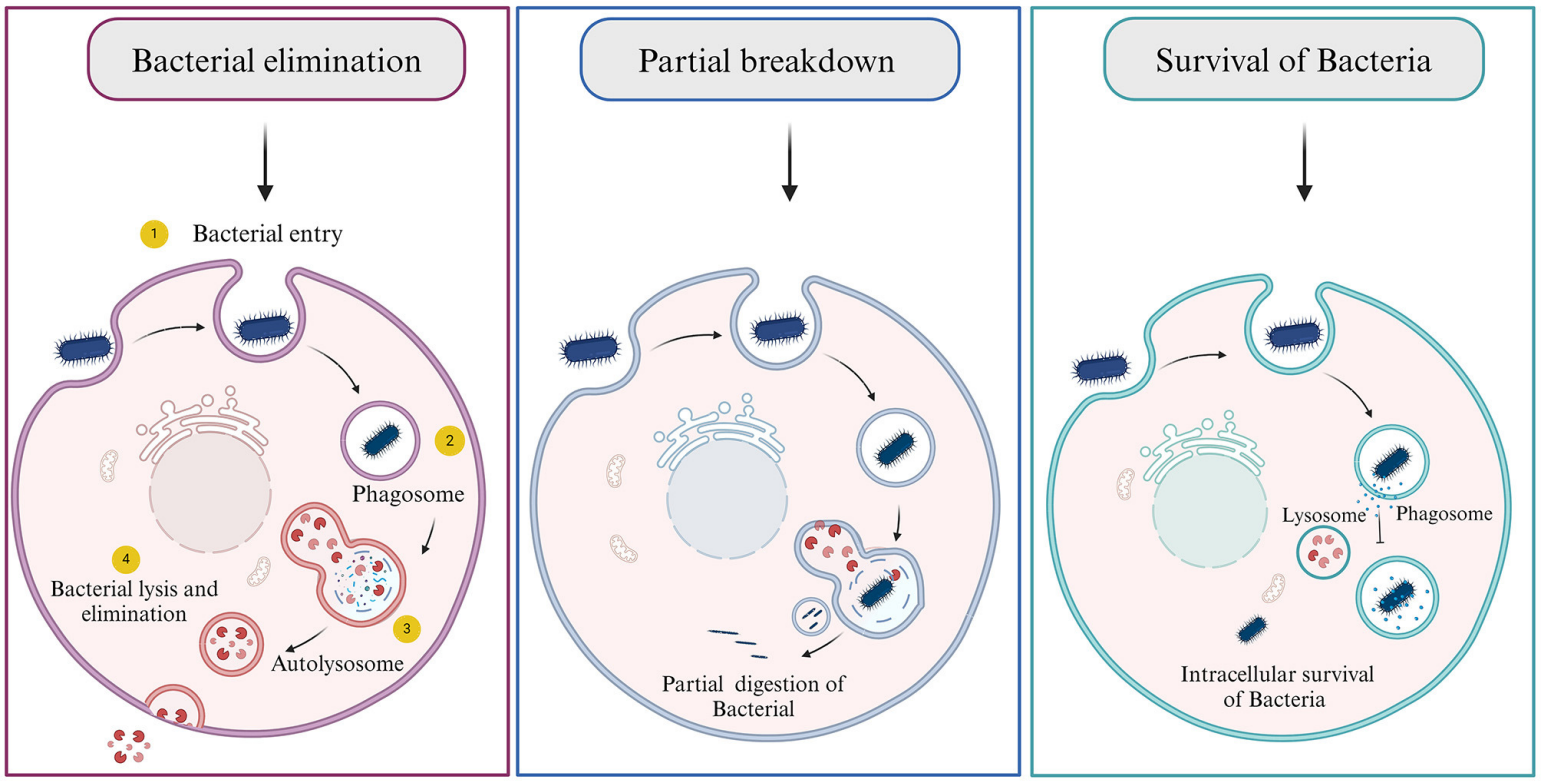
Consequences of bacterial infection. (1) Bacterial elimination by host cell, (2) Partial digestion of bacteria, and (3) bacterial escape and survival.

When bacteria target a host cell and choose to invade it for shelter, they employ several evasion strategies to circumvent or neutralize the host's robust immune defenses (Hornef et al., 2002) There are three possibilities arose as the host cell encounters bacteria: (1) the killing/degradation of bacteria by autophagolysosomal fusion, (2) partial digestion of bacteria where the genome of bacteria can persist inside the cell for a longer time and may or may not cause any harm, and (3) where the bacteria successfully evade and survive inside the host cells (Figure 5) (Asrat et al., 2014; Choi et al., 2018; Riebisch et al., 2021). These methods include secreting proteins that can degrade or hinder the host's immune system, modifying the surface of their own membranes, or imitating the actions of host factors. This intricate interplay allows them to establish residence within the host and ensure their safety.

To manage the indigenous colonizing microflora and counteract pathogens, the human body has developed a diverse array of host defense mechanisms. These strategies encompass physical aspects, such as the skin and mucosal linings, mechanical elements like ciliated cells within the respiratory tracts and tight junctions, and biochemical defenses present in bodily fluids like tears and saliva, which contain the antimicrobial enzyme lysozyme (Janeway et al., 2001). These multifaceted defenses are remarkably proficient at preventing the emergence of invasive microbial diseases. Furthermore, the body possesses two adaptable immune defense systems, namely the innate and adaptive immune systems, which can be mobilized as needed (Hilchie et al., 2013).

The innate immune response, the first line of host defense found in all multicellular organisms, predates the acquired immune response. It has evolved to protect the host from a range of toxins and infectious agents like bacteria, fungi, viruses, and parasites. Innate immunity involves preexisting mechanisms like physical barriers, enzymes (e.g., lysozyme), phagocytes, inflammation-related proteins (e.g., complement proteins), antimicrobial peptides, cell receptors (e.g., Toll-like receptors), professional antigen presenting cells, and cells releasing cytokines (Rousset, 2023). However, some bacteria target the protective cells like macrophages (*Listeria monocytogenes*), dendritic cells (*Coxiella burnetii*), and neutrophils (*Chlamydia pneumoniae*). These cells act as a reservoir for many microbes which successfully thrive intracellularly (Gorvel et al., 2014; Mitchell et al., 2016; Kobayashi et al., 2018). It acts rapidly in minutes to hours to prevent infection and initiate the acquired immune response when a new pathogen is encountered. This natural response aims to prevent infection, eliminate invaders, and trigger the adaptive immune response. The innate immune cells recognize bacteria by identifying PAMP-pathogen distinct molecular patterns through certain receptors known as PRRs (pathogen recognition receptors). These receptors can be further classified into various classes, with TLRs (toll-like receptors) being one of the most common. Once these receptors bind with PAMPs, they set off several immune signaling pathways that are necessary for an early infection response and trigger the adaptive immune system to help further control the pathogens. The innate immune response doesn't possess immunological memory and is incapable of recognizing a previously encountered pathogen upon subsequent exposure. Wherein, adaptive immunity is antigen-specific, and takes some time between antigen exposure and the maximal response (Mogensen, 2009).

The adaptive immune response involves identifying specific “foreign” antigens, differentiating them from “self” antigens, initiating pathogen-specific immunologic effectors designed for the precise elimination of pathogens or infected cells, and nurturing immunological memory to enable swift eradication of a particular pathogen upon potential reinfections. Adaptive immunity involves a complex network of interactions among B lymphocytes, T lymphocytes, and antigen-presenting cells. B cells perform dual function as they differentiate into plasma cells that produce antibodies along with the production of memory B cells upon activation by foreign invaders. Secondly, they also act as antigen-presenting cell and activates T cells by assisting recognition of a specific antigen, Bacterial pathogens causing infections are transported to macrophages or dendritic cells, influencing the activation of pathogen-specific T cells. On the other hand, pathogens confined to infected tissues may initiate T cell activation through soluble factors, thus impeding a range of host virulence factors (Shepherd and McLaren, 2020). Bacterial virulence factors modulate the immune synapse, aiding the bacteria in evading the immune mechanisms of host cells. This unique aspect of adaptive immunity lies in its ability to establish a memory, enabling a swifter and more efficient immune response upon re-exposure to the antigen (Capitani and Baldari, 2022).

#### Mechanisms of bacterial immune evasion

i. *Modulation of surface molecules*: Bacteria have several surface features that are critical for immune recognition, including lipid A, flagella, and peptidoglycans. However, bacterial pathogens, particularly gram-negative bacteria, have evolved methods to evade immune detection by altering these molecules. For example, Salmonella uses a two-component sensor to modify lipid A, thereby reduce TLR4 activation. Some bacteria, such as *Porphyromonas gingivalis*, produce diverse lipid A species that selectively moderate inflammatory responses. Peptidoglycan, another essential bacterial molecule, is detected by the intracellular proteins Nod1 and Nod2, triggering an inflammatory response. Pathogens have developed methods to avoid peptidoglycan recognition, and genes involved in its synthesis have been identified as virulence factors. For example, Listeria monocytogenes uses surface-located peptidoglycan hydrolases as virulence factors to exploit Nod2 and promote pathogenesis (Leite Pereira et al., 2020).
ii. *Antigenic variation in bacteria*: Bacterial pathogens, such as *Neisseria* species that cause meningitis and gonorrhea, employ diverse mechanisms for antigenic variation. For instance*, Neisseria*, which causes meningitis and gonorrhea, exhibits antigenic variation through diverse molecular mechanisms. These include multiple copies with independent switches, one expression locus with silent gene copies, and a variable region in the molecules. The constant changes in antigenic molecules, particularly in *Neisseria*, challenge vaccine development and contribute to their ability to survive within the host (Deitsch et al., 2009).
iii. *Subversion of phagocytic cells*: Bacterial pathogens employ diverse strategies to evade killing by phagocytic cells. Internally, pathogens use tactics, such as escape from the phagosome, inhibition of phagosome-lysosome fusion, or survival in phagolysosomes. Some bacteria such as *Shigella* and *Listeria*, secrete lysins to breach vacuolar membranes. Intracellular pathogens manipulate vesicular trafficking. Legionella uses a type IV secretion system, and *Salmonella* employs a Spi-2 type III secretion system and *M. tuberculosis* prevents phagosome acidification. Pathogens modulate inflammatory responses; some activate pathways (e.g., *Shigella* and *Salmonella* induce caspase-1 activation), whereas others dampen inflammation (e.g., Yersinia with YopJ targeting the MAP kinase and NFκB pathways) (Sarantis and Grinstein, 2012).
iv. *Bacterial subversion in innate and adaptive pathways*: Bacterial pathogens employ various strategies to evade the innate immune response. For instance, Yersinia species secrete a virulence factor, inducing immunosuppression through CD-14 and TLR2-dependent signaling, resulting in IL-10 secretion. *Salmonella* adapts to cationic peptides by modifying lipid A, expressing the PgtE protease, and employing a peptide transport locus (sapA-F), all of which are coordinated by the global regulator PhoP/Q. *Salmonella* pathogenicity island 2 (Spi2) shields intracellular Salmonella from reactive nitrogen intermediates, thereby avoiding co-localization with iNOS and NADPH oxidase-mediated killing. Collectively, these mechanisms enable bacteria to circumvent immediate immune responses and establish infection (Baxt et al., 2013).

Bacterial pathogens can also modulate adaptive immune responses. Helicobacter pylori interferes with immune cells by binding to C-type lectin receptors on dendritic cells, resulting in a mixed Th1/Th2 response. The mixed Th1/Th2 response may have implications for the host's ability to combat infection and could contribute to the persistence of the bacteria. Similarly, *N. gonorrhoeae* Opa proteins bind to CD4+ T cells and suppress their activation. *Streptococcal* superantigens alter T-cell distribution and affect disease severity. Mucosal pathogens, such as *Neisseria* and *Haemophilus*, secrete IgA proteases, degrade mucosal antibodies, and impair immune defense.

## The challenge of antimicrobial resistance

Bacterial infections pose a significant global burden, with increasing drug resistance exacerbating the problem. Multidrug-resistant bacterial infections are becoming increasingly common, leading to longer hospital stays, increased use of expensive antibiotics, and higher morbidity and mortality rates. Specific examples of bacterial infections with severe consequences include salmonellosis, tuberculosis, and cholera, which collectively cause millions of cases and thousands of deaths each year. *Salmonella enterica serovar Typhimurium* causes ~93.8 million cases of salmonellosis annually, with a death toll of up to 150,000 cases (Galán-Relaño et al., 2023). We have also included specific examples under the subsection of antimicrobial resistance. With the introduction of antibiotics, it was initially believed that infection rates would decrease. However, bacteria have demonstrated their ability to outsmart antibiotics, leading to what is commonly known as the antibiotic crisis. As many bacteria have evolved to exhibit antimicrobial resistance, this phenomenon has substantial implications for infectious diseases, ultimately favoring the survival of these resilient bacteria (Michael et al., 2014). Antimicrobial agents target various bacterial mechanisms and processes, including: (1) Inhibition of cell wall synthesis, (2) disruption of protein synthesis, (3) impairment of nucleic acid synthesis, and (4) interference with metabolic pathways. This adaptability of bacteria poses a considerable challenge in the battle against infectious diseases (Reygaert, 2018).

One huge conundrum of antimicrobial resistance is that the use of these drugs leads to increased resistance. Even the use of low or very low concentrations of antimicrobials can lead to selection of high-level resistance in successive bacterial generations, may select for bacteria that are hypermutable strains (increase the mutation rate), may increase the ability to acquire resistance to other antimicrobial agents, and may promote the movement of mobile genetic elements. Bacteria develop resistance through genetic transfer and mutations, often involving plasmids. Stressors like UV radiation can induce mutations, but most are detrimental (Martinez and Baquero, 2000). Resistance mutations mainly impact drug targets, transporters, and modifying enzymes, often reducing the organism's growth rate. Paradoxically, antimicrobial use can boost resistance, even at low levels, promoting hypermutability, cross-resistance, and genetic element transfer.

Antimicrobial resistance mechanisms can be categorized into four main groups:

- *Restricting drug entry*: Bacteria can become antibiotic-resistant by changing their outer membrane's permeability, which serves as a protective barrier. This outer membrane contains protein channels called porins, allowing the entry of substances, including antibiotics. Bacteria can develop resistance by modifying these porins to restrict antibiotic influx. This modification acts as a defense strategy, making it harder for antibiotics to enter the bacterial cell and exert their effects, enabling bacteria to survive in the presence of antibiotics.
- *Altering drug targets*: A successful bacterial strategy against antibiotics involves producing enzymes that either modify the drug or synthesis enzymes that can induce chemical modifications in antimicrobial molecules.
- *Deactivating drugs*: Another strategy involves addition of chemical groups or completely break down the antibiotic, rendering it ineffective in targeting its intended site.
- *Actively expelling drugs*: Bacteria employ efflux pumps as a mechanism to develop antibiotic resistance. These pumps are categorized into various classes, and they can be found in both gram-negative and gram-positive pathogens. Some of these pumps are substrate-specific, designed to expel antibiotics, like the *tet* (tetracycline) determinants for tetracycline and *mef* (macrolides in pneumococci) genes for macrolides in pneumococci. Others have broad substrate specificity and are often associated with multidrug-resistant (MDR) bacteria. Efflux pumps impact a wide range of antimicrobial classes, including inhibitors of protein synthesis, fluoroquinolones, β-lactams, carbapenems, and polymyxins. The genes responsible for encoding these efflux pumps can be in mobile genetic elements (MGEs) or within the bacterial chromosome (Munita and Arias, 2016).

Intrinsic resistance may involve limiting drug entry, deactivating drugs, and drug expulsion, while acquired resistance mechanisms encompass altering drug targets, deactivating drugs, and drug expulsion.

## Pathogenic bacteria and their implications in infectious diseases

Various bacterial pathogens are resistant to antimicrobial agents. This increase in antimicrobial resistance among pathogenic bacteria has rendered the treatment of certain common infections, such as pneumonia, exceedingly challenging and, in some cases, nearly impossible. To address this threat to human health, the World Health Organization (WHO) compiled a list of priority pathogens, including *Enterococcus faecium, Staphylococcus aureus, Klebsiella pneumoniae, Acinetobacter baumannii, Pseudomonas aeruginosa*, and *Enterobacter* spp., collectively known as ESKAPE. These pathogens alone are responsible for causing >50% of the infection related bacterial deaths globally.

i. *Acinetobacter baumannii* is the most lethal bacterium in bloodstream infections. It exhibits resistance mechanisms such as the production of beta-lactamase, efflux pumps, and enzymatic modification of aminoglycosides and modified porins (Tamma et al., 2022).
ii. *Pseudomonas aeruginosa* demonstrates intrinsic resistance mechanisms, including efflux pump overexpression, reduced outer membrane permeability, and acquired or mutated resistance genes.
iii. *Staphylococcus aureus* rapidly develops antibiotic resistance, including methicillin- and vancomycin-resistant strains.
iv. *Klebsiella pneumoniae* exhibits high antibiotic resistance due to the acquisition of genes encoding enzymes like carbapenemases.
v. *Enterobacter* spp. *and Enterococci* also display various resistance mechanisms. Furthermore, *Escherichia coli (E. coli)* acquires resistance genes through horizontal gene transfer, extended-spectrum β-lactamases, carbapenemases, and plasmid-mediated quinolone resistance genes.

The significant resistance observed in these bacterial pathogens further challenges the treatment of infectious diseases. With the limited advancement for antibiotic therapies and the emergence of multidrug-resistant strains, there is an urgent need to develop new antimicrobial strategies. The threat of antimicrobial resistance not only compromises the ability to combat infections but also presents severe challenges for vulnerable patient populations undergoing medical treatments. As resistance trends persist, it is imperative to explore alternative therapies, invest in drug development, and implement stringent antibiotic stewardship to effectively address the implications of antibiotic-resistant bacterial pathogens (Mulani et al., 2019).

## Strategies for combating antimicrobial resistance

- *Drug development*: The increasing prevalence of antimicrobial resistance (AMR) mandates an ongoing quest for novel drug candidates to effectively combat infections. Delving into the mechanisms of AMR not only provides guidance but also functions as a valuable tool in the development for new drugs. Furthermore, the absence of swift and dependable diagnostics has led to inappropriate antibiotic prescriptions in clinical settings, resulting in increased antibiotic exposure and the hastening of resistance development (De Rycker et al., 2018). To tackle the challenge of drug development in the context of AMR, one potential solution is the exploration of diverse genetic interactions to create innovative drug combinations against antibiotic-resistant bacteria. Recognizing that chemical-genetic signatures are unique to each species offers an opportunity to expedite the creation of specific drug combinations. These precisely targeted approaches can amplify the efficacy of antibiotics and enhance AMR management (Silver, 2022).
- *Vaccine development*: AMR is a severe global health issue. Developing vaccines targeting resistant pathogens is a promising solution. To combat antimicrobial resistance, vaccines are a crucial weapon. The pathogens that cause infections are directly inhibited from spreading by a vaccine. Reducing this spread lowers the overall number of infections and lowers the likelihood that a pathogen will evolve into a form that is resistant to drugs. Research into pathogenesis and immune responses informs vaccine design. New adjuvants can enhance protein-based vaccine effectiveness. Simple and cost-effective older vaccine technologies, like live attenuated and inactivated vaccines, are viable. Presently, cost-effectiveness analyses often overlook AMR's impact on vaccine value. To combat this threat to global health, increasing vaccination coverage, enhancing efficacy, and accounting for AMR effects in evaluations are essential steps (Micoli et al., 2021).

## Bacterial pathogenicity and its impact on public health

The presence and pathogenicity of bacteria in healthcare units pose significant challenges to public health, affecting both communities and healthcare systems in various ways. Bacterial infections have a large impact on public health and can be transmitted through physical contact, air, water, food, or living vectors. The impact of bacterial pathogenicity on public health includes the transmission of pathogenic bacteria from healthcare setups which contributes to increased morbidity and mortality amongst the immune-compromised patients (Peacock and Newton, 2008). The treatment of these infections requires extended hospital stays, specialized medications, and intensive care, contributing to elevated healthcare costs. Medical infrastructures like hospitals, clinics, and primary health centers serve as potential reservoirs for broader community transmission.

Preventive measures, such as water treatment, immunization, personal hygiene, infection prevention, and control in healthcare settings, have a dramatic impact on reducing morbidity and mortality. Bacteria, such as *Mycobacterium tuberculosis* contribute to the global burden of diseases, straining healthcare resources. *Helicobacter pylori'*s impact on the stomach is linked to various gastrointestinal diseases, affecting community health. Gram-positive bacteria (e.g., *Staphylococcus aureus*, and *Streptococcus pneumoniae*) cause widespread illnesses, impacting both individuals and healthcare infrastructure. *Pseudomonas aeruginosa* infections pose challenges in healthcare settings, demanding extensive resources for patient care (GBD 2019 Antimicrobial Resistance Collaborators, 2022).

### Implications for healthcare systems

Prioritizing epidemiologic surveillance allows for effective monitoring and control of infections, aiding early detection and outbreak management. Healthcare workers face increased occupational health risks, emphasizing the need for stringent protocols including regular cleaning and safety guidelines. Establishing and adhering to these measures mitigates the impact of bacterial contamination on both patients and healthcare personnel. Identifying the prevalent bacteria in healthcare units would enable the prioritization of public health actions, including tailored protocols and guidelines. Community education through healthcare systems minimizes the spread of infections beyond healthcare units and promotes responsible health practices.

## Limitations and potential shortcomings

Diverse arrays of bacteria and their distinct immune evasion and survival strategies present a formidable challenge for comprehensive coverage in a single review. Each bacterial species exhibits unique methods for evading the host immune system, making comprehensive coverage in a single review a formidable task. Temporal considerations and the dynamic nature of bacterial pathogens pose complexities. The increasing prevalence of antimicrobial resistance (AMR) further adds on a layer of complexity that influences our understanding of well-known bacterial pathogens and their resistance patterns to specific drugs. Exploring bacterial pathogens at the molecular level poses technological challenges, whereas the intricate environmental conditions crucial for bacterial behavior are challenging to control. Moreover, the variability in host response to bacterial infections, driven by genetic, immune, and health dissimilarity, further complicates the understanding of bacterial pathogenesis.

## Conclusion

Bacterial pathogens exhibit remarkable diversity in colonizing various niches within the human body and employing sophisticated strategies for survival and replication. The severity of infection depends on multiple factors, of which immune evasion is of paramount importance. These evasion mechanisms include inhibition of immune signaling pathways, internalize within host cells, disrupting phagosomes, deactivating reactive oxygen species, and modulating the host immune response. Host susceptibility, which is also influenced by genetic polymorphisms, introduces significant variability in the type and intensity of responses to the encountered pathogens.

The rapid emergence of antibiotic resistance poses a formidable challenge. The inherent ability of bacteria to resist antibiotic exposure exacerbates genetic changes caused by inappropriate antibiotic practices. The overuse of antibiotics not only eliminates susceptible bacteria but also facilitates the proliferation of drug-resistant strains. Factors like insufficient sanitation, poor infection control practices, and the widespread application of antibiotics in animal husbandry add to the alarming surge of antimicrobial resistance. Resolving this critical problem necessitates a comprehensive strategy that includes judicious antibiotic usage, better sanitation practices, and increased awareness of the effects of antimicrobial resistance on public health.

While this review provides an overview of bacterial pathogenesis and the emerging antimicrobial resistance crisis, there is a pressing need for further research and in-depth knowledge to enhance our understanding of bacterial pathogens. Future research on bacterial pathogenesis and infections should focus on investigating new antibiotic resistance mechanisms, particularly in bacteria that are evolving, like *E. coli*. Exploring horizontal gene transfer dynamics and understanding zoonotic transmission can help prevent the spread of resistance genes. Embracing a wholistic “One Health” approach that integrates human, animal, and environmental health is crucial for addressing the complexity of bacterial infections. Investing in the development of new drugs and antibiotics, comprehending host-pathogen interactions, and building rapid diagnostic tools are crucial aspects of effective intervention strategies.

## Author contributions

RP: Conceptualization, Funding acquisition, Project administration, Supervision, Visualization, Writing – review & editing. JS: Data curation, Investigation, Visualization, Writing – original draft. SS: Investigation, Resources, Writing – original draft.
